# Supplementary material for: Essential Role of COP9 Signalosome Subunit 5 (Csn5) in Insect Pathogenicity and Asexual Development of Beauveria bassiana
Source: J Fungi (Basel). 2021 Aug 7;7(8):642. doi: 10.3390/jof7080642 (PMC8401740; doi:10.3390/jof7080642)
Supplement: Supplementary file 1 [file jof-07-00642-s001.zip › jof-1307227-supplementary.pdf]

Supplementary Material

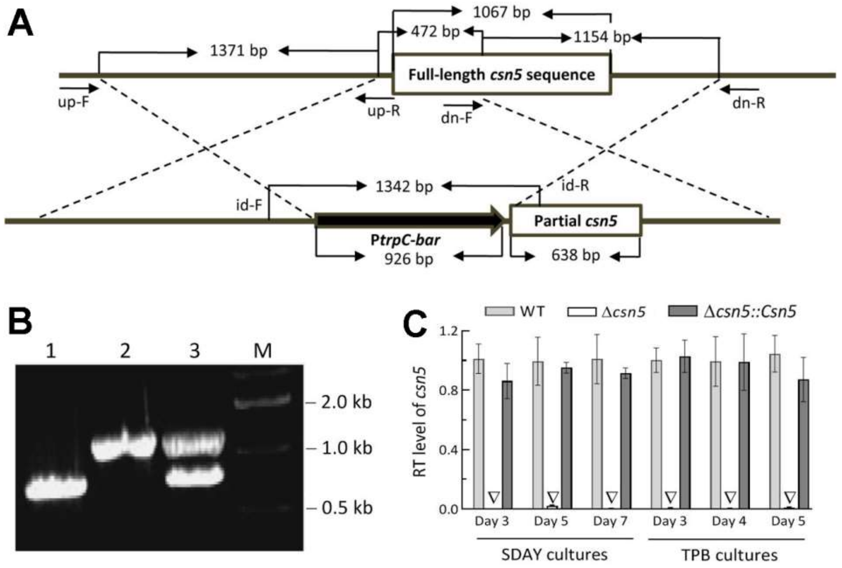

Figure S1. Generation and identification of *csn5* mutants in *B. bassiana*. (**A**) Schematic diagram for the disruption strategy of *csn5*. (**B**) The *csn5* mutants identified through PCR analysis (lanes 1–3: WT, Δ*csn5* and Δ*csn5::Csn5* respectively; M, DNA ladder) with paired primers in Table S1. The detected PCR bands denote the fragments of 888 bp in Δ*csn5*, 1,342 bp in WT and both of them in Δ*csn5::Csn5* (888 + 926 − 1342 = 472 bp), indicating that *Csn5* was disrupted by the deletion of its promoter/coding fragment (43 + 429 = 472 bp) as expected. (**C**) Relative transcript (RT) levels of *Csn5* in the SDAY and insect hemolymph-mimicking TPB cultures of *csn5* mutants with respect to the WT standard during 7- and 5-day incubation at optimal 25°C respectively. Note that expression of *Csn5* was abolished in the Δ*csn5* cultures and restored in the Δ*csn5::Csn5* cultures. Error bars: standard deviations of the means from three cDNA samples derived from independent cultures of each strain.

Table S1. Paired primers designed for manipulation of *csn5* in *B. bassiana.*

Primers	Paired sequences (5′–3′)*	Purpose
Csn5-F/R	CAATCACAAACACCTTCAAAATGGAAACGGCTCTCAGGTCATG / CTCGCCCTTGCTC ACCATTGTACTGTTTTGATCCCCCAAGTCG	Cloning *csn5* cDNA (1014 bp) for fusion to *gfp*
upCsn5-F/R	ACGAGCTGTACAAGTAACCCGGGGAGCCTATGCGGTATCTATG / TGGCTGCAGGTC GACGGATCCGAGTTGGGAGTTAGTGTTGC	Cloning *csn5* 5' fragment (1371 bp)
dnCsn5-F/R	GACCCATGGCTCGAGTCTAGAAAAATGTTGTCGGATGGTA / GGTGGTGGTGGCTAG CGTTAACTTCTGTCAGTGGAGGATTG	Cloning *csn5* 3' fragment (1154 bp)
flCsn5-F/R	ATCCGTCGACCTGCAGCCAAGCTTAAACCAAGCAGGTAAGGC / ACACTAGTCAGAT CTTCTAGTGTACACCCACGACATTCAGG	Cloning full-length *csn5* for complementation (3991 bp)
pCsn5-F/R	CTCCAGCATTCGCCAACA / CTGAAACCCATCCGCACC	PCR detecting *csn5* (888 bp)
qCsn5-F/R	TCGTGACTTTGCAAACAAGC / GACGCCAGTAGCCAGAAATC	qPCR detecting *csn5*

* Underlined regions denote introduced cleavage sites of two pairs of restriction enzymes (*Xma*I/*Bam*HI and *Xba*I/*Hpa*I) for homogeneous recombination of the *bar*-separated 5′ and 3′ fragments for the disruption of *csn5* or the fragments for gateway exchange.

Table S2. Paired primers used for transcriptional profiling of *csn5*-related genes in *B. bassiana*.

Gene	Tag locus [a]	Annotation [b]	Sequences (5′–3′) of paired primers
**Used as internal standard**			
act	BBA_04860	β-actin	GGCAACATTGTCATGTCTGG / TTTGCTGGAAGGTGGATAGG
**Involved in asexual development and hydrophobicity**			
brlA	BBA_07544	Developmental activator BrlA	GATGGATGACAAGTGCGATG / AAACTCGCACGAGAAACGAT
abaA	BBA_00300	Developmental activator AbaA	GCAAGTCTCCAGCCATAT / CTCCTCTTCGTCATAGTAGTC
wetA	BBA_06126	Developmental activator WetA	ATGCGGTACTACAGCCAAGG / GAGTTCCTGCTGGCTACTGG
vosA	BBA_01023	The velvet protein VosA	ACTCATGGGCTCATTGGTGG / CCGGCAAGAGAGATCCGAAA
hyd1	BBA_03015	Class I hydrophobin Hyd1	ATGGTGGAAAGGATCTGCAC / TGGGAAAGAAGACCATCAGC
hyd2	BBA_06599	Class II hydrophobin Hyd2	TGTCAAGACTGGCGACATTT / ATTGGGACAAGCTGGTTGAG
**Involved in response to heat shock**			
hsp30a	BBA_08688	Heat shock protein 30	AGGCGTGAAAAAGGAAGACA / GGTTGACGCCTCTGTCGTAT
hsp30b	BBA_02057	Heat shock protein 30	ATTGCTCGCAACAACAACAA / CAAGATACCGTCCTGGAAGC
hsp40a	BBA_06930	Chaperone DnaJ, Mdj1	AAACCCTGGCGATGATACTG / AGAAACGGCTGAAAAGGTCA
hsp40b	BBA_08440	Heat shock protein, Hsp40, DnaJ	CAAGAAGTGCAAGGGAAAGC/ TACCGAGACGCGAAAAGACT
hsp40c	BBA_03736	Chaperone protein dnaJ 2, Mas5	CAAGAAGTGCAAGGGAAAGC/ TACCGAGACGCGAAAAGACT
hsp60	BBA_05467	Chaperonin GroL	AGAAGAACAAGCGCGACATT/ GGTGATGAAGTAGGGCGAAA
hsp70a	BBA_00941	Hsp70 protein	ACGCTTGAAAGTCTGCCCTA / GGATTTCATACGAGCCTGGA
hsp70b	BBA_05586	Heat shock protein 70-2	CACGTACTCTTGCGTCGGTA / ACCTCGGGGTCTGAGAACTT
hsp90	BBA_06516	Heat shock protein 90	GGTCAGTTCGGTGTTGGTTT / CGTCCTTGAGGTGGAGGATA
hsp104	BBA_02283	Heat shock protein HSP98	ATGTTCCCGACAACCTGAAG / GCACCCATGAGAAGGTGAAT
**Involved in ubiquitin activation (E1 family)**			
ubi1/2	BBA_06852	Ubiquitin family protein	GCCAAGCTGCTAGCATTACC / CGCCATGAAAAGGAAAACAT
ubi3	BBA_09309	Ubiquitin family protein	ATCCTCCCCACCATTACCTC / CTGGGTCGTCCTTGTCTAGC
ubi4	BBA_03881	Polyubiquitin precursor Ubi4	GCTCCTCTTTAGCAGCATGG / GCAAAACTGCACGTAATCCA
**Involved in ubiquitin conjugation (E2 family)**			
ubc1	BBA_07096	E2 ubiquitin-conjugating protein UBC1	CGATAGCTACCCGTTCAAGC / GGGTTGCTCGTTCATCATCT
ubc2	BBA_01469	E2 ubiquitin-conjugating protein RAD6	GTGACTTCAAGCGAATGCAA / GCTCCTCAAACTGCATCACA
ubc3	BBA_06665	E2 ubiquitin-conjugating enzyme	GGACGAGCGAGAAAAGTACG / TCCATGTCACTGTCGCTACC
ubc4	BBA_01937	E2 ubiquitin-conjugating protein UBC4	ATTGTGGGACCTCACGAGAC / ACCTCTCCACGTGCCTAGAA
ubc5	BBA_09007	E2 ubiquitin-conjugating protein UBC5	TCTACCACCCAAACGTCACA / TCTCCCATGGCGTACTTTTC
ubc6	BBA_03114	E2 ubiquitin-conjugating protein UBC6	AATATTCCCGCCCAACTACC / ACATTCGTTGAGACGGAACC
ubc7	BBA_02005	E2 ubiquitin-conjugating protein UBC7	GAGCTCAAGTTTCCCAAGGA / GAATCTTTTCGACCGACTGG
ubc8	BBA_06237	E2 ubiquitin-conjugating protein UBC8	TCGCCAATGTTTGACATGAT / ATCGTCGTCATCTTCGCTCT
ubc9	BBA_06098	E2 SUMO-conjugating protein UBC9	TACAGCAAGAGCGCAAGCTA / GGGTATTCATCGGGGAAAGT
ubc10	BBA_00143	E2 ubiquitin-protein ligase peroxin 4	GTCGTCCAACGTCTCCAAAT / CACTGCATCAAGCTCTCGTC
ubc11	BBA_00216	E2 ubiquitin-protein ligase UBC11	AGCTTGCAGACGGAACTGAT / GGGTAGTTGGAGGGGAAGTC
ubc12	BBA_04666	NEDD8-conjugating protein UBC12	ACCTTTGCCATCAACCAGAG / GGCTCGAGGAACAAAAACTG
ubc13	BBA_09651	E2 ubiquitin-conjugating protein UBC13	ACGAAGGTGGCATTTTCAAG / GGGGACCAGTTGTTTTTCAA
ubc14	BBA_07061	Ubiquitin-conjugating protein DMA2	GCTGGGAGGAGTTGTGTCAT / TTGGGTTCCTTGGCAGTTAC

Table S2 (continued)

Gene	Tag locus [a]	Annotation [b]	Sequences (5′–3′) of paired primers
**Involved in ubiquitin-protein ligation (E3 family)**			
upl1	BBA_00743	Ubiquitin-protein ligase TUL1	GTTTCGCCTTTGTCATCCTC / GCAAAGTCTCCCCATTGTGT
upl2	BBA_00804	RBR-type E3 ubiquitin transferase	ACGTCTTTGGCACCATTAGG / GTCCCCGTAAAAGTCGCATA
upl3	BBA_01028	E3 ubiquitin-protein ligase BRE1	AGGGGAAGTTAAACCCGAGA / TGCTGTTCCAACTTCTGCAC
upl4	BBA_01431	E3 ubiquitin-protein ligase HEL1	GCCTGCTTTGCTGATAGTCC / GTTCGCAACCAAGAGAAAGC
upl5	BBA_02443	E3 ubiquitin-protein ligase PRP19	TTGATGCCTCACTCACAACC / CTGCTAGCCAGAAACCGTTC
upl6	BBA_03508	E3 ubiquitin-protein ligase RAD18	CTGCGGAGTAATTGGTCCAT / CTACACTCGGCGTAGGTGGT
upl7	BBA_04432	E3 ubiquitin-protein ligase UBR1	GCGCTAACTAGGGCATCAAG / ACCAAGGTGGATAAGCGATG
upl8	BBA_05204	E3 ubiquitin-protein ligase HEL1	CTCGCCGAGTCCAACTACTC / CCAGCGTTTCGTAGAGTTCC
upl9	BBA_05776	E3 ubiquitin-protein ligase HEL2	GGAGCAGCATTTCAAAAAGG / GAGGTCAAAACCAGCCATGT
upl10	BBA_06925	Ubiquitin-protein ligase peroxin 12	TTTCGAGTCTGTCGGTAGCC / TAAATGCTTGGCCCAAAGAC
upl11	BBA_07173	E3 ubiquitin-protein ligase IRC20	CTTTTAGTGCGGTCGAGGAG / ATCGGCCGTTCTTTATTGTG
upl12	BBA_07231	E3 ubiquitin-protein ligase HRD1	GCAAGAAGTCAGGGAACAGC / TCCAGCCAAAGTACCCATTC
upl13	BBA_07605	E3 ubiquitin-protein ligase RKR1	CGAGGCCAAGTACTTTCGAG / CATGGCCTGTCTGCACTCTA
upl14	BBA_09149	E3 ubiquitin-protein ligase SSM4	GCGCGAAGAATTTGAGGTAG / GGCGAATTCATGTACGTGTG
upl15	BBA_09504	Ubiquitin-protein ligase PSH1	ATTGCGGAACAACATCAACA / TGACGATCGCCTTCTCTCTT
upl16	BBA_01822	NEDD4 family E3 ubiquitin-protein ligase	TGGTGAGCAAAAGTCACGTC / GTATGTGCCTTGGGCAAGTT
upl17	BBA_03558	Putative ubiquitin-protein ligase UFD4	ATCAGCTCATAAGCCGCAGT / AGACGCAAGATGGAAGCTGT
upl18	BBA_05494	Putative E3 ubiquitin-protein ligase HUL4	GTCCGGAAGAGATTGAGCTG / TCACTGCCGGTGATAAATGA
upl19	BBA_05518	E3 ubiquitin-protein ligase TOM1	GCGAACTCATTACCGAGAGC / CCACTTGTTTGTCCCTCGTT
upl20	BBA_05644	Ubiquitin-ubiquitin ligase HUL5	CTCTGGCCTCAGAACACACA / CTCCAGGCACGGTTTGTAAT
upl21	BBA_00267	Ubiquitin ligase complex subunit HRD3	AGCTTGTCGATTTGGCCTTG / CAGGTATAGTGCGTCCGAGT
upl22	BBA_03528	Putative ubiquitin-protein ligase ASI3	TTGCATGCACATCCGAACAA / AGAGCGGCCACATAGTAGAC
upl23	BBA_05538	Ub ligase molybdopterin-converting factor	GACGCCATTGACAACATCGA / CAGCCCATCGTCGGTACTAT
**Involved in deubiquitination**			
usp1	BBA_02705	Ubiquitin-specific protease UBP1	GAGCGTGCACTACGAGATGA / ACAGATCGCTGTCCGAGAGT
usp2	BBA_07124	Ubiquitin-specific protease UBP2	AGCGATCTTCAGGCACAGAT / ATCAAAGCTGACCTCGTGCT
usp3	BBA_08453	Ubiquitin-specific protease UBP3	ACTCTTTGCCAAGACGCCTA / GCCAACCTTGTAGGATCGAA
usp4	BBA_04102	Putative ubiquitin-specific protease UBP12	CTCTTGACCGCAAGGTTCTC / GGGCAAGTCAATCAGCAAAT
usp5	BBA_00871	Ubiquitin-specific protease YUH1	CGGAGATTCGCTTCAACCTG / TTCTCCTCTCCAGACTCCCA
usp6	BBA_01234	Ubiquitin-specific protease YUH1	CTTCACATCGCTTCTCCACG / CGGCGGTCTTGTTGTAAGTC
usp7	BBA_01266	Ubiquitin-specific protease YUH1	GCAATAAGAGGCGCAATCCA / GCACCTTGACGTCCTTGATC
usp8	BBA_01860	Ubiquitin-specific protease UBP15	GCGACAAACTACGGCTACTG / GTAATCAGCCGCGACACAAT
usp9	BBA_02115	Ubiquitin-specific protease UBP13	CAACGCCAGGCTATCAACTC / TCCTTGAGCCCGGTAAATGT
usp10	BBA_02323	Ubiquitin-specific protease YUH1	GTCCAACGTGAATCCTGACC / GGGATAAAAGCGAGCAGGTC
usp11	BBA_03889	Ubiquitin-specific protease UBP13	CCAAGCTCAACAAGGCCATT / CGTAAATGGAGCGCGATAGG
usp12	BBA_04237	Ubiquitin-specific protease UBP11	CCAAGCTCAACAAGGCCATT / CGTAAATGGAGCGCGATAGG
usp13	BBA_04750	Ubiquitin-specific protease UBP15	CTCGGCAAGAAGGAACATGG / AACGAGGGCAAACTGAACAC
usp14	BBA_04857	Ubiquitin-specific protease UBP14	TCCCCAGCCATTCATCAGTT / CCACCATAGAGCCTTTTCGC
usp15	BBA_06326	Ubiquitin-specific protease UBP14	TTCTGACGCCAACTCTGCTA / TGAACAACCACTCAACAGCG
usp16	BBA_07695	Ubiquitin-specific protease UBP6	CTTTACACGAGGATGAGCGC / CGCCGCTCATGTACTTTTGA
usp17	BBA_10345	Ubiquitin-specific protease UBP8	CCAGCGTCACCTATGTTTGG / TTTGCGGCCAGAGAAAGAAC

[a] Gene accession codes of *B. bassiana* genome under the NCBI accession NL_ADAH00000000.

[b] Putative E2, E3 and UBP genes are annotated with respect to their corresponding homologs in budding yeast.

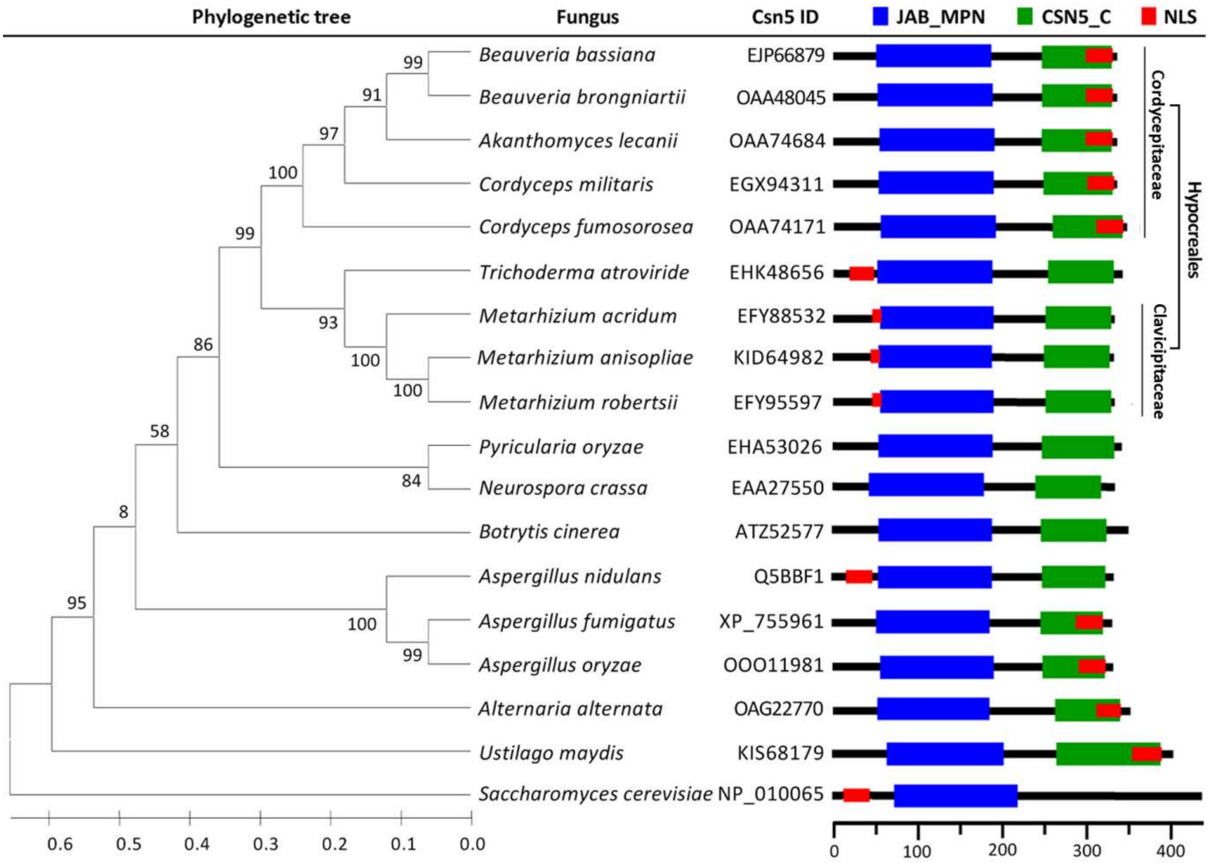

Figure S2. Phylogenetic relationships and sequence features of Csn5 orthologues found in entomopathogenic (Hypocreales) and nonentomopathogenic fungi. Scale for phylogenetic tree: branch length proportional to genetic distance assessed with the maximum likelihood method in MEGA7 at http://www.megasoftware.net/. Bootstrap values of 1000 replications are shown at nodes. Main domains and NLS motif are predicted at https://www.ncbi.nlm.nih.gov/Structure/ and http://nls-mapper.iab.keio.ac.jp/ , respectively.

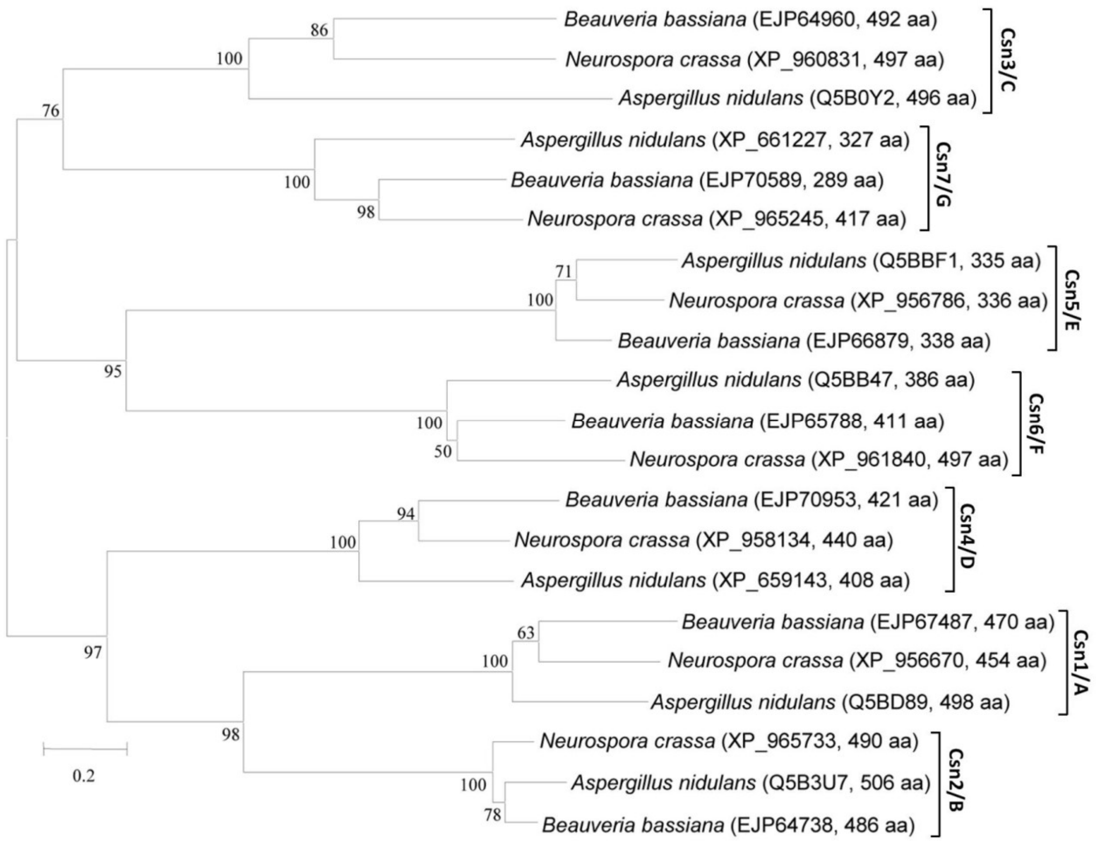

Figure S3. Phylogenetic relationships of CSN subunits between *B. bassiana* and two model fungi. Scale: branch length proportional to the genetic distance assessed with the maximum likelihood method in MEGA7 at http://www.megasoftware.net/. Bootstrap values of 1000 replications are shown at nodes. The NCBI accession code of each subunit and the length of its amino acid sequence are listed in the parentheses following the fungal name.

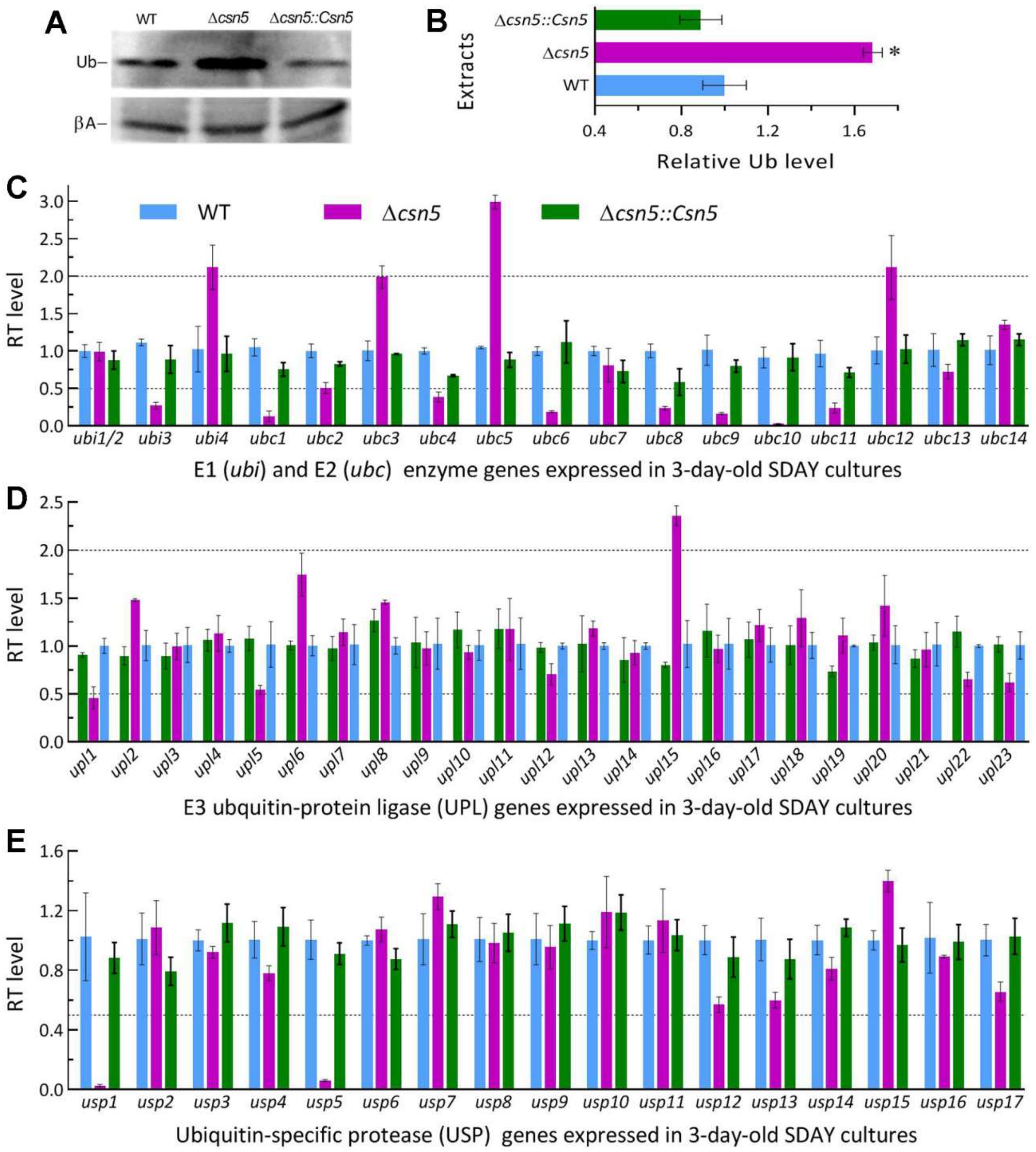

Figure S4. Impact of *csn5* deletion on accumulation level of intracellular free ubiquitin and expression levels of putative UPS genes in *B. bassiana*. (**A, B**) Western blot for free ubiquitin (Ub) accumulation probed with monoclonal anti-Ub antibody in the protein extracts of 3-day-old SDBY cultures and relative Ub accumulation levels computed as ratios of Ub blot intensities over those of β-actin (βA) as a reference probed with anti-βA antibodies, respectively. Each lane was uploaded with 20 μg of protein sample. **P* < 0.001 in Tukey's HSD test. (**C–E**) Relative transcript (RT) levels of E1, E2, E3 and ubiquitin-specific protease (USP) genes in the 3-day-old SDAY cultures of *csn5* mutants with respect to the WT standard. The dashed lines indicate a significant level of one-fold change (50% downregulation or 100% upregulation relative to WT). Error bars: standard deviations of the means from three independent replicates.
